# Non‐Pulmonary Vein Substrate Ablation of Recurrent Atrial Fibrillation in the Patient With Fabry Disease

**DOI:** 10.1002/ccr3.70945

**Published:** 2025-09-21

**Authors:** Koumei Onuki, Kenichi Hiroshima, Akihiro Isotani, Masato Fukunaga, Michio Nagashima, Kenji Ando

**Affiliations:** ^1^ Department of Cardiology Kokura Memorial Hospital Kitakyushu Japan

**Keywords:** atrial fibrillation, Fabry disease, fractionated signal areas of the atrial muscle, pulmonary vein isolation

## Abstract

Fabry disease (FD) is a rare disease that progressively causes myocardial degeneration. There are only a few low‐voltage areas, but some fractionated potentials are present in the left atrium of FD patients with atrial fibrillation (AF). Attempting non‐pulmonary vein (non‐PV) substrate ablation may be worthwhile.

## 1 Introduction

FD is an X‐linked lysosomal storage disorder caused by mutations in the GLA gene, leading to a deficiency of lysosomal α‐galactosidase A (α‐Gal A). Classic FD results in multiorgan failure, while the later‐onset phenotype is characterized predominantly by cardiac manifestations. Due to its rarity, there are few reports on arrhythmia treatment in patients with the later‐onset phenotype of FD. Regarding AF ablation in patients with FD, it remains unclear whether PVI alone is sufficient. On the other hand, high‐resolution mapping obtained with recently developed mapping catheters allows for the visualization of various functional abnormalities. We report a case in which high‐resolution mapping and non‐PV substrate ablation were performed for recurrent AF in a patient with FD.

## 2 Case History/Examination

**FIGURE 1:**
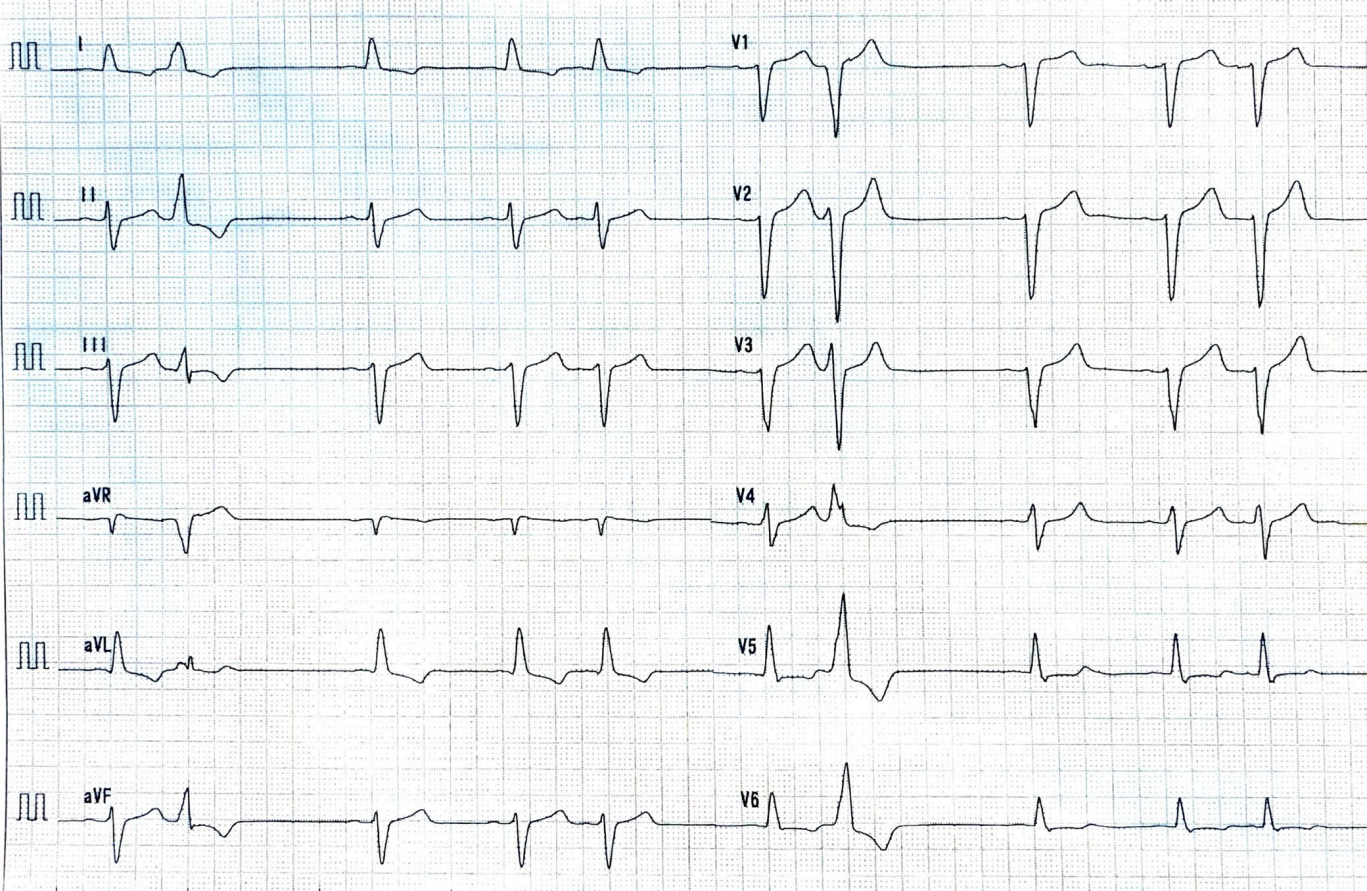
Baseline electrocardiogram.

The patient was a 73‐year‐old man. In year X−11, extensive encircling PVI and a cavo‐tricuspid isthmus (CTI) block line were performed to treat AF and atrial flutter. During provocation testing with isoproterenol infusion and intravenous adenosine, atrial fibrillation was induced by atrial premature contraction firing from the right superior pulmonary vein (RSPV); accordingly, the RSPV was determined to be the arrhythmogenic vein. No abnormalities were observed in the atrial potentials or substrate, so line ablation and substrate ablation were not performed in the first session. At that time, the patient had mild left ventricular hypertrophy (LVH) with a wall thickness of 12.7 mm, and magnetic resonance imaging (MRI) was not performed. In year X−1, heart failure exacerbation occurred despite optimal pharmacologic therapy at another institution. Subsequently, heart failure symptoms improved to NYHA class I. In year X, the patient experienced a recurrence of paroxysmal AF. Baseline electrocardiogram (ECG) is shown in Figure 1.

On general examination, he was conscious, cooperative, and well oriented to time, place, and person. The vital signs were stable; pulse rate was 60 beats/min, irregular, with normal volume and character; blood pressure was 112/62 mm of Hg in bilateral upper limbs; jugular venous pressure was normal; and respiratory rate was 12 breaths per min. There was no edema in the lower legs, and cardiac auscultation revealed the presence of a third heart sound (S3). No corneal opacity was observed, and both visual acuity and visual fields were normal. Neither hypohidrosis nor anhidrosis was present, and no significant gastrointestinal symptoms were noted.

## 3 Differential Diagnosis, Investigation, and Treatment

As differential diagnoses for LVH, hypertrophic cardiomyopathy, hypertensive heart disease, and FD were considered. Preoperative transthoracic echocardiography (TTE) showed an enlarged left atrial diameter of 54 mm and diffuse LVH with a wall thickness of 14.4 mm was observed. The ejection fraction (EF) was 44%, with globally hypokinetic wall motion. MRI revealed linear late gadolinium enhancement (LGE) in the basal ventricular septum, with faint enhancement also observed in other ventricular walls and atrial walls. Due to the significant reduction in α‐Gal A activity to below 1% and characteristic findings on TTE, including LVH and diastolic dysfunction, a definitive diagnosis of FD was made without awaiting genetic test results. Mulberry bodies were detected in the urine. Genetic testing confirmed a c.644A>G (p.N215S) mutation in Exon 5. Based on these findings, a definitive diagnosis of the later‐onset phenotype of FD was made.

**FIGURE 2:**
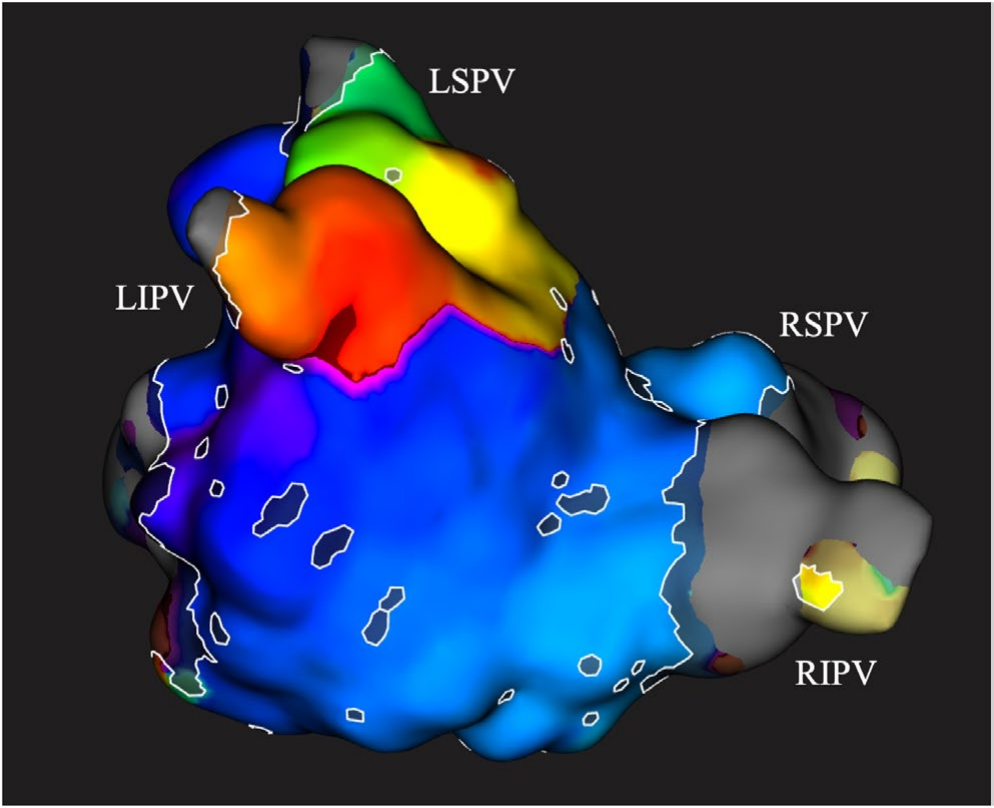
Left atrium activation map. The activation map is shown. The view is posterior–anterior, looking upward from below. Reconnection was confirmed from the bottom of the LIPV. LIPV, left inferior pulmonary vein; LSPV, left superior pulmonary vein; RIPV, right inferior pulmonary vein; RSPV, right superior pulmonary vein.

**FIGURE 3:**
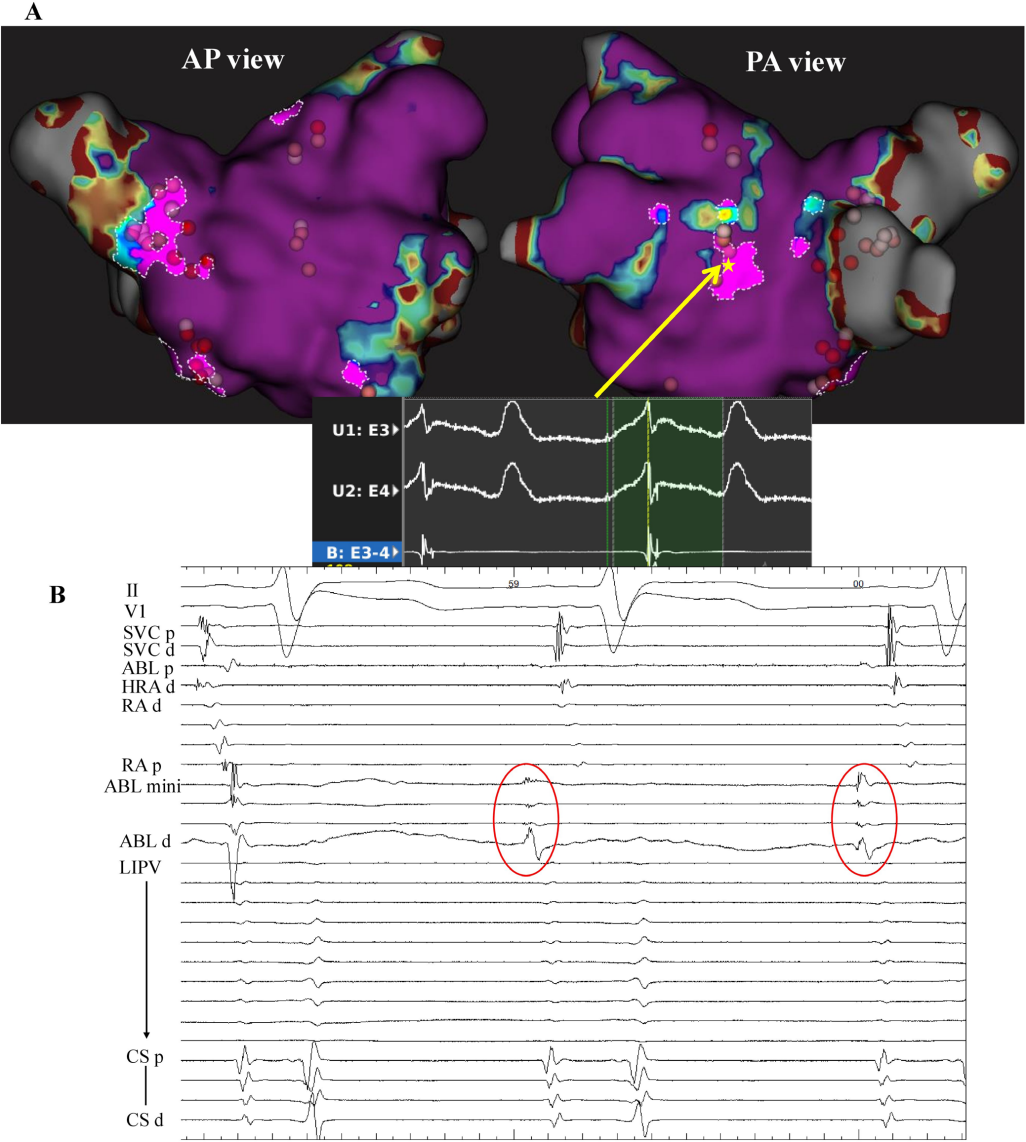
FAAM map with ablation tag and intracardiac electrogram of APCs. The areas of FAAM7 in the left atrium and the ablation tags (red tags) in the AP and PA views are shown in (A). Frequent APCs were observed from the fractionated signal areas on the posterior left atrial wall (asterisked location). Unipolar and bipolar potentials of the mapping catheter are shown at the bottom of the (A). And the intracardiac electrogram is shown in (B). Frequent APCs were observed, with the earliest activation detected on the mini electrodes of the ablation catheter. ABL mini, Mini electrodes of the ablation catheter; ABL, ablation catheter; AP, anterior–posterior; APCs, atrial premature contractions; CS, coronary sinus; d, distal; FAAM, fractionated signal areas of the atrial muscle; HRA, high right atrium; *p*, proximal; PA, posterior–anterior; RA, right atrium; SVC, superior vena cava, and other abbreviations were similar to that of Figures 1 and 2.

The patient underwent a second catheter ablation due to symptomatic and drug‐resistant AF. The ablation session was performed under deep sedation. Activation mapping of the left and right atrium during sinus rhythm was conducted using a 64‐pole mini‐basket catheter with the RHYTHMIA mapping system (Boston Scientific, Marlborough, Massachusetts). The left atrial volume was 237.33 cc. Reconnection of the left inferior pulmonary vein (LIPV), which had not been identified as an arrhythmogenic vein in the first session, was observed, and re‐isolation was performed (Figure 2). Under isoproterenol (1–10 μg/kg per minute), bolus injection of adenosine triphosphate was used to induce AF. Even after re‐isolation of the LIPV, atrial burst pacing easily induced AF. Following induction, frequent APCs were observed in the fractionated signal areas on the left atrial posterior wall (Figure 3B). To prevent the formation of an isthmus, the fractionated potentials were compartmentalized by applying short‐duration, low‐power ablation lesions (25–30 W for 10–15 s). After ablation of the left atrial posterior wall, APCs were significantly reduced, and AF could no longer be sustained, even with ganglionated plexi stimulation. Subsequently, residual fractionated potentials that were not identified as arrhythmia origins—specifically those anterior to the RSPV and on the left atrial septum—were similarly ablated. The final ablation sites are indicated by the red tags in Figure 3A. In the right atrium, no fractionated potentials were observed. The procedure was completed without complications. The patient was discharged in stable condition.

## 4 Outcome and Follow‐Up

Follow‐up was conducted with outpatient visits every one to two months to monitor symptoms and ECG findings. The patient has remained free from any episodes of recurrent tachycardia for a year. Fortunately, this patient had not developed fibrosis or LVA within the left atrium, and even MRI showed no prominent LGE in the atrium. Enzyme replacement therapy has proven useful for preventing further disease progression.

## 5 Discussion

Cardiac involvement in FD is common and results from the accumulation of glycoproteins within myocytes, vascular endothelium, valves, and conduction tissues. FD is not associated with decreased QRS complex amplitude but rather shows ECG features of LV hypertrophy commensurate with the wall thickness [1]. A pattern of pre‐excitation can be seen. However, in some cases, when deposition occurs within the atrioventricular node, the PR interval may be prolonged [2]. In atrial arrhythmias with FD patients, one report found that 3.9% of patients with FD had persistent AF, while 13.3% had paroxysmal AF [3]. The incidence is likely to be higher, with numerous mechanisms in FD contributing to the structural, functional, and electrical substrate that is required for AF to initiate and persist. However, reports specifically addressing AF in FD patients are limited. Most previous studies have focused instead on ventricular arrhythmia, pacemaker implantation, and atrial tachycardia, leaving a gap in information on managing AF in FD patients [3, 4, 5, 6]. This scarcity of data is due to the small number of patients and the variable phenotypic expression of the disease. There has been one previous report on ablation for recurrent AF in a patient with FD [7]. In that case, PVI was performed twice and was reported to be effective in maintaining sinus rhythm in a patient with classic FD. However, as this investigation was limited to a single case with a short follow‐up period of only six months, it remains unclear whether PVI alone is sufficient for managing AF in patients with FD. To the best of our knowledge, this is the first report of non‐PV substrate ablation for recurrent AF with FD.

For substrate ablation in AF, techniques such as low‐voltage area (LVA) ablation have been utilized, though no standardized approach has been established. Masuda et al. identified substrates posing a risk for recurrent AF using high‐resolution mapping [8]. In their report, independent risk factors for AF recurrence included not only LVA (< 1.0 mV, > 5 cm^2^) but also fractionated‐electrogram areas (≥ 5 peaks, > 5 cm^2^). Therefore, modifying the fractionated area as a substrate ablation for AF recurrence patients with minimal LVA may be reasonable. And we have previously focused on fractionated signal areas and reported that the fractionated signal areas of the atrial muscle (FAAM) ablation are useful in identifying non‐PV foci, with this technique showing favorable outcomes even at the one‐year follow‐up [9, 10]. The Lumipoint software (Rhythmia system, Boston Scientific) provided dedicated visualization, highlighting the FAAM areas. In the current case of AF recurrence with no LVA, substrate ablation was performed targeting the FAAM areas, resulting in favorable outcomes.

Patients with FD tolerate arrhythmia, supraventricular tachycardia, and AF poorly due to impaired diastolic filling. Therefore, rhythm control through ablation and other methods is the favored strategy. Although there is now general support for early ablation in all patients presenting with AF, there remain concerns regarding long‐term efficacy in FD [11]. This is because long‐term success is compromised by progressive atrial dilatation, impaired atrial function, and changes in atrial structure. Managing atrial fibrillation may be useful not only for preventing cardiac death but also for reducing the risk of stroke; further experience is needed for AF treatment in patients with FD.

Finally, we employ a continuous infusion of isoproterenol (1–10 μg/kg/min) combined with bolus injections of adenosine triphosphate to determine whether it possesses arrhythmogenicity. In addition, reproducible atrial premature contractions observed upon electrical cardioversion are designated as ablation targets [12]. This strategy is supported by evidence indicating that the elimination of all AF foci is important [13]. Therefore, we define non‐inducibility of AF as the procedural endpoint. In this case, reconnection of the LIPV was observed, but it lacked arrhythmogenicity, and PVI alone did not reduce the inducibility or persistence of AF. FD is a rare disease that progressively causes myocardial degeneration; therefore, it may be necessary to adopt a treatment strategy different from that used for ablation in typical AF patients. On MRI, LGE was observed throughout the atrial myocardium, suggesting diffuse accumulation of α‐Gal A in the entire myocardium. In the treatment of AF in FD patients with advanced myocardial involvement, as in this case, PVI alone may be insufficient. Attempting non‐PV substrate ablation in AF patients with FD may be worthwhile. Further experiences are needed.

## Author Contributions

**Koumei Onuki:** conceptualization, investigation, visualization, writing – original draft. **Kenichi Hiroshima:** formal analysis, writing – review and editing. **Akihiro Isotani:** formal analysis, writing – review and editing. **Masato Fukunaga:** formal analysis, supervision. **Michio Nagashima:** formal analysis, supervision. **Kenji Ando:** formal analysis, supervision.

## Consent

Written consent was obtained from the mother of the patient after death.

## Conflicts of Interest

The authors declare no conflicts of interest.

## Data Availability

Data supporting the findings of this study is available from the corresponding author on reasonable request.
